# Exosomes as Mediators of Cell-to-Cell Communication in Thyroid Disease

**DOI:** 10.1155/2020/4378345

**Published:** 2020-04-28

**Authors:** Yi Wang, Feng Xu, Jia-Yu Zhong, Xiao Lin, Su-Kang Shan, Bei Guo, Ming-Hui Zheng, Ling-Qing Yuan

**Affiliations:** ^1^Department of Metabolism and Endocrinology, National Clinical Research Center for Metabolic Diseases, Hunan Provincial Key Laboratory of Metabolic Bone Diseases, The Second Xiang-Ya Hospital, Central South University, Changsha, Hunan, China; ^2^Department of Radiology, The Second Xiang-Ya Hospital, Central South University, Changsha, Hunan, China

## Abstract

Exosomes are a type of extracellular vehicle, formed by budding cell membranes, containing proteins, DNA, and RNA. Concentrated cargoes from parent cells are enveloped in exosomes, which are cell specific and may have functions in the recipient cell, reflecting a novel physiological and pathological mechanism in disease development. As a transmitter, exosomes shuttle to different cells or tissues and mediate communications among these organelles. To date, several studies have demonstrated that exosomes play crucial roles in disease pathogenesis and development, such as breast and prostate cancer. However, studies investigating connections between exosomes and thyroid disease are limited. In this review, recent research advances on exosomes in thyroid cancer and Graves' disease are reviewed. These studies suggest that exosomes are involved in thyroid disease and appear as impressive potentials in thyroid therapeutic areas.

## 1. Exosomes

**Figure 1:**
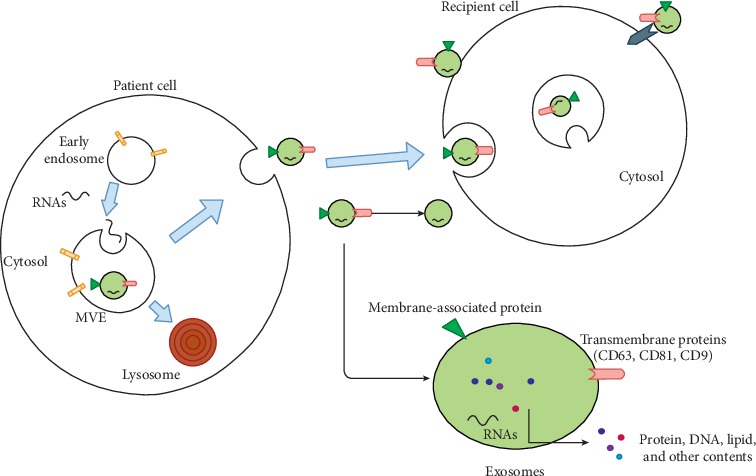
The formation and transmitting of exosomes. Exosomes are formed as the vehicles by budding into multivesicular endosomes (MVEs) and then released by the fusion of MVEs with the plasma membrane. Other MVEs proceed into lysosome pathway for degradation. Exosomes released from patient cells transmitting to recipient cells affect recipient cells by direct fusion with membrane of recipient cells, endocytic uptake, and ligand-mediated interaction. CD63, CD81, and CD9 are common surface biomarkers of exosomes. RNAs, protein, DNA, and other contents encased into exosomes can be transmitted into target cells.

Exosomes are extracellular vehicles (EVs) with approximate sizes of 40–100 nm with a density of 1.13–1.19 g/ml in sucrose density gradients; formed by a process of endosome membrane intracellular invagination [1], exosomes are released from patient cell, transmitting into circulation and specific sites (Figure 1). In 1987, these membrane vesicles were initially observed in rat reticulocytes [2] and termed “exosomes” [3]. Exosome cargoes are varied, including lipids [4], DNA [5], proteins [6], growth factors [7], and several types of RNAs [8–10], such as microRNA (miRNA), long noncoding RNA (lncRNA), circular RNA (circRNA), and mRNA. The mechanism of those cytosolic components selectively transferred into exosomes is not revealed clearly. Recently, the endosomal proteins such as Hsc70 and Hsp90 could potentially play a role in exosomal protein sorting [9]. Furthermore, the endosomal sorting complex responsible for transport (ESCRT)-II and specific sequence motifs shared by RNAs in exosomes may be associated with RNA sorting [10, 11]. It had been exhibited that exosomes virtually exist in all bodily fluids, including serum, plasma, and urine [12–14].

### 1.1. The Function of Exosomes

Diverse exosomal functions have been described, chiefly including serving as transmitters shuttling between different cells and mediating intracellular communications, such as cellular differentiation, promoting angiogenesis, and modulating immune responses [15–20]. These functions can be divided into two types: disease development and disease reversal.

Exosome cargoes, when targeted to recipient cells, can behave as disease contributors. A crucial role of exosomes in HIV infection revealed recently [21]. Chiozzini et al. observed that when dendritic cells (DC) mediated the trans-dissemination of exosomes from HIV-1-infected cells to resting CD4^+^ T lymphocytes, the process induced efficient trans-dissemination and HIV-1 expression in target cells [22]. Yi et al. observed that miR-146a in exosomes suppressed type I interferon responses and facilitated EV71 infection [23]. Ye et al. uncovered that exosomes derived from celecoxib on nasopharyngeal carcinoma TW03 cells generated T-cell dysfunction, by inhibiting T-cell proliferation and Th1 and Th17 differentiation and promoting Treg induction in nasopharyngeal carcinoma cells [24]. These results suggest that exosomes secreted from parental cells have impact on the function of immune cells, especially T cells.

Angiogenesis plays an important role in oncogenesis and metastasis. Kosaka et al. showed that upregulation of miRNA-351b and miRNA-210 in exosomes released by cancer cells, resulted in enhanced tumor angiogenesis [25]. Hsu et al. observed that miRNA-23a in exosomes derived from lung cancer cells increased tumor angiogenesis under hypoxic and normal conditions [26]. These observations show that miRNAs in exosomes secreted from cancer cells enhance angiogenesis, promoting tumor development in cancer.

Proteins harboured in exosomes appeared to facilitate disease procedures as well. Blomme et al. showed that myoferlin was overexpressed in exosomes derived from different breast and pancreatic cancer cell lines, promoting human umbilical vein endothelial cell (HUVEC) migration and invasion. In contrast, myoferlin-depleted exosomes exhibited decreased abilities in inducing HUVEC proliferation and migration [27]. Additionally, proteins attached to exosome cell surfaces contribute to exosomal functions. Christianson et al. showed that heparan sulfate proteoglycans (HSPGs) function as internalizing receptors of exosomes, inhibiting exosome-mediated migration of cancer cells through proteoglycans in proteoglycan-deficient cells [28]. These data demonstrate that proteins in exosomes derived from cancer cells, directly or indirectly, affect tumor migration.

Similarly, exosomes function in disease resistance. Exosome cargoes can act as mitigating factors in several molecular scenarios. Zhao et al. found that exosomes containing active signal transducers and activators of transcription 3 (STAT3) produced by adipose-derived stem cells could be transferred into macrophages, inducing anti-inflammatory M2 phenotypes through transactivation of arginase-1 [29]. Song et al. revealed that miRNA-146, a well-known anti-inflammatory factor, packaged in exosomes derived from mesenchymal stem cells, targeted macrophages, resulting in anti-inflammatory M2 phenotype formation [30]. It appears that anti-inflammatory M2 phenotypes generated by exosomal mechanisms are efficient in assisting inflammatory disorder reversal. Exosomes play a role in cardiovascular system as well. Our present study investigated that exosomes derived from melatonin-treated vascular smooth muscle cells could mitigate vascular calcification and aging through an exosomal miR-204/miR-211 cluste [31]. Cheng et al. proposed a mechanism whereby miRNAs enveloped in impaired myocardium-derived exosomes can be selectively transferred into bone marrow cells to mobilise progenitor cell migration to lesions, promoting heart tissue repair [30]. These discoveries indicate a key property of exosomes in damage resistance and healing acceleration at damaged sites. In conclusion, exosomes contribute to an enhanced interpretation of cell physiology and pathology.

## 2. Thyroid Diseases

Thyroid diseases can be broadly categorized as hypothyroidism, hyperthyroidism, goiter, and cancers. Both hyperthyroidism and hypothyroidism are thyroid dysfunctions, presenting symptomatic and readily diagnosed by laboratory data of thyroid hormones, which can be treated under further investigation of clear and definite causes. The most clinically important thyroid diseases are thyroid cancer (TC) and thyroid goiter. As the most prevalent malignancy of the endocrine system, TC represents approximately 1.5% to 2.1% of all cancers diagnosed annually worldwide, being divided into differentiated carcinoma, which includes papillary thyroid cancer (PTC), follicular and Hurthle cell cancer [33], and undifferentiated carcinoma, which consists of medullary thyroid cancer (MTC) and anaplastic thyroid cancer (ATC) [34]. PTC accounts for 80–85% of all TCs. According to clinical statistics, young females are the main targets for PTC [35]. Even though comparatively satisfactory PTC prognosticated in the clinic, a small portion of PTC turns aggressive, meaning the tumor is capable of developing into distant metastases, thereby increasing the death rate [36]. With the highest mortality rate, ATC exists as the most aggressive tumor of these four types [37]. Up to now, the main problem with TC treatment was early and overdiagnosis, as well as neoplasm staging, all of which could be resolved to a large extent by exosome biology.

Thyroid goiter comprises nontoxic or toxic conditions, bringing inconvenience to patients and lowering their quality of life. Reports have recommended that globally goiter is mainly caused by iodine deficiency, while several other factors give rise to thyroid goiter as well, such as multinodular thyroid disease, chronic autoimmune thyroiditis, and Graves' disease (GD) [38, 39]. GD deserves further research as it seriously impacts the quality of life.

## 3. Exosomes in TCs

Even as a common endocrine malignancy, the specific pathogenesis/tumorigenesis of TC remains unclear. Up to now, more than a few TC mechanisms have been elucidated, involving several signalling pathways, such as the mitogen-activated protein kinases (MAPK) pathway, and the phosphatidylinositol-3-kinase and protein kinase B (PI3K–AKT) pathway, and molecular rearrangements such as mutation, gene copy-number gain, and aberrant gene methylation [40]. It had been proposed that cargoes harboured in exosomes are connected to signalling pathways. Additionally, explorative connections between exosomes biology and cancers, such as breast, prostate, ovarian, and lung cancer, are ongoing. Similarly, the relationship between exosomes and thyroid disease has lately emerged; therefore, do exosomes play biological roles in thyroid-related diseases, and furthermore could exosomes perform potential functions in thyroid disease therapy?

### 3.1. Variation in Expression of Exosomal Cargoes in TC

Exosomes function in cell-to-cell communications, including normal cells to cancer cells. Several studies have revealed that exosome cargos are alterable as well as cargo quantities depend on the disease process. It appears that cargo up- or downregulation makes crucial differences to biological outcomes.

Lee et al. proposed that PTC cell line cell-derived exosomes contained overexpressed miRNA-146b and miRNA-222 molecules, when compared with normal thyroid cells. Furthermore, reductions of meaningful fold of these two miRNAs levels after total thyroidectomy were detected in the plasma, correspondingly [41]. Roman et al. showed that miRNA-31-5p, miRNA-146a-5p, miRNA-21-5p, miRNA-221-3p, and miRNA-181a-5p were statistically overexpressed in PTC-derived exosomes. Precisely, overrepresented miRNA-31-5p was found when compared with benign tumors while miRNA-146a-5p, miRNA-21-5p, miRNA-221-3p, and miRNA-181a-5p were increased corresponding to other TC types [42]. Similarly, another study found that miRNA-21 and miRNA-181a in PTC-derived exosomes were differentially expressed. Yang et al. revealed that 22 circular RNAs (circRNAs), in serum exosomes derived from PTC patients, were differentially expressed, when processed by high-throughput sequencing. Compared with the control group, three circRNAs including hsacirc_007293, hsacirc_031752, and hsacirc_020135 were upregulated, and 19 were downregulated [43]. Wang et al. demonstrated that miR-346, miR-10a-5p, and miR-34a-5p were upregulated in PTC-derived exosomes [44]. Differential expression of miRNAs in exosomes derived from PTC patients may indicate different functions in PTC pathogenesis. Variations in mechanisms and pathways have been investigated. According to DIANA-Tarbase analysis, miR-346 may be associated with several pathways, including the TC p53 signalling pathway, mammalian target of rapamycin (mTOR) signalling pathway, and the MAPK signalling pathway. MiR-10a-5p was found to interact with cell cycle, p53, and MAPK signalling pathways in TC pathogenesis [44]. Majority information was focused on the variation of miRNA, whereas few detections concerned with rest of substances encapsulated within exosomes had been discovered. Edo et al. proposed that exosomes expressing the thyrotropin receptor (TSHR) may be secreted from normal and cancerous thyroid cells [45]. Luo et al. found 697 differentially expressed proteins in serum-purified exosomes (SPEs) of PTC patients with lymph node metastasis (LNM), when compared with SPEs from PTC patients without LNM [46]. While mechanisms have not been fully revealed, these observations provide clues that exosomal cargoes are intentionally delivered into selected cells. Further studies are required to investigate exosomal cargoes and their significance in pathophysiology.

### 3.2. The Role of Exosomal Cargoes in TC Development

**Figure 2:**
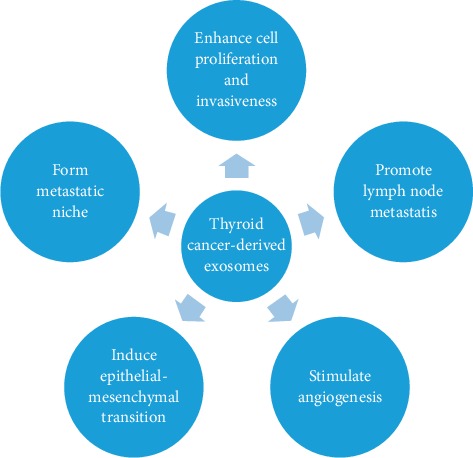
The role of TC-derived exosomes in cancer development. Exosomes derived from thyroid cancer cells promote the development of TC by direct and indirect ways. Direct approaches include enhance cell proliferation and invasiveness and promote lymph node metastasis. Indirect approach includes form metastatic niche, stimulate angiogenesis, and induce epithelial-mesenchymal transition to support tumor progress.

As the differential expression of exosomal cargoes has been proposed, further studies revealed their significance and mechanism. It has been suggested that exosomes derived from original cells, containing certain molecules, can transport these cargoes to target cells near and far, thereby causing tumor development. Several exosomal mechanisms have been proposed for the development of TC (Figure 2).

Luo et al. revealed that SRC, TLN1, ITGB2, and CAPNS1 proteins were overexpressed in PTC-derived exosomes, accelerating tumor metastasis. Furthermore, integrin-associated proteins were upregulated in SPE from PTC with LNM, when compared with patients without LNM [46]. Our recent study suggested that exosomes isolated from hypoxic PTC, BCPAP, and KTC-1 cells contained increased miRNA-21-5p levels, which contributed to HUVEC angiogenesis. Angiogenesis was attenuated after the knockdown of miRNA-21-5p in exosomes. Our study revealed a new mechanism of hypoxic PTC angiogenesis, via the exosomal miRNA-21-5p/TGFBI and miRNA-21-5p/COL4A1 pathways, suggesting an original perspective for PTC therapy [47]. Ye et al. demonstrated that exosomal miRNA-432-5p in PTC serum exosomes was significantly overexpressed and associated with lymph node metastasis; the capacity for PTC cell migration and invasion was enhanced by exosomal miRNA-432-5p [48]. These results suggest that protein and miRNA levels in exosomes are associated with TC stage and LNM, which hit the disease procedure and prognosis.

Epithelial-mesenchymal transitions (EMT) occur under the influence of exosomal cargoes as well. The EMT involves epithelial cells that undergo a transition to a mesenchymal phenotype, characterised by the loss of E-cadherin, which is activated by factors such as SNAIL, SLUG, ZEBs and TWIST [36]. Cells gain the ability to transfer and invade other cells, promoting the metastasis of primary tumours to develop into secondary tumours [36]. Studies have suggested that in thyroid tumours, the induction of EMT was associated with the overexpression of vimentin, related to PTC. Additionally, the invasion and lymph node metastasis of PTC occurs through EMT [49]. The loss of E-cadherin and membranous *β*-catenin, and the activation of SLUG and TWIST mechanisms are linked to undifferentiated carcinomas, especially ATCs [50, 51].

A study found that thyroid cancer stem-like cell (CSC) derived exosomes, transferred lncRNA, specifically linc-ROR, to trigger EMT, inducing local tumor microenvironments and distant metastatic niches [36]. In contrast, exosomal miRNA-145 suppressed the growth and transfer of TC cells; Compared with the healthy group, the expression of exosomal miR-145 was significantly decreased in TCs. Additionally, the upregulation of miR-145 in TC cells triggered decreased cell proliferation, migration, invasion, vascular endothelial growth factor (VEGF) secretion and E-cadherin expression, by targeting the PI3K/Akt pathway [52]. In conclusion, the function of exosomal contents can be seen in Table 1.

### 3.3. Exosomes as Fluid Biopsy in TC Diagnosis and Therapy

In different pathological states and stages, abundant exosomes encapsulating different substances exist in the circulation. Most of these variations are corresponding with the pathological stages, suggesting analysis of the differential expression of these cargoes may provide novel outlooks on TC diagnosis and therapy.

#### 3.3.1. Exosomal-Mediated Therapies

Because endosomes appear to differentially promote disease development and disease resistance, their potential exploitation as disease treatments has aroused the interest of many researchers. Further exploratory studies have focused on therapeutic areas, where exosomes act as delivery tools to transport drugs to targeted areas. Several studies concerning exosome therapeutic effects in cancer diseases [53, 54], inflammatory diseases [53], and even in psychological illness [56] emerged.

Hoshino et al. found that tumor-secreted exosomes, which was uptaken by organ-specific cells to prepare the premetastatic niche, played an important role in tumor metastasis. Also, specific exosomal integrin expression pattern was related to different tumor metastases [55]. Richards et al. revealed treatment of cancer-associated fibroblasts with an inhibitor of exosome biogenesis GW4869 attenuated chemoresistance in cocultured cancer cell *in vitro* and inhibited cancer growth *in vivo* [56]. These observations imply that blocking exosome biogenesis and secretion could be a feasible approach for disease therapy.

In order to deliver targeted drug, Qi et al. developed a exosome-based superparamagnetic nanoparticle cluster which can serves as drug-transmitting vehicles. The results showed that reticulocyte-derived doxorubicin-loaded exosomes could facilitate cancer targeting in an external magnetic field and suppress tumor growth [57]. Additionally, Xiao et al. suggested that tumor exosome-loaded dendritic cells could activate and recruit effector T cells, and prolong the survival time of pancreatic cancer patients, when combined with cytotoxic drug treatments [58]. These results suggest that exogenous molecules can be carried by exosomes and transferred to targeted areas.

#### 3.3.2. Fluid Biopsy in TC

Fluid biopsy is a novel concept which consists of circulating tumor DNA (ctDNA), circulating tumor cells (CTCs), and exosomes [59]. There are several drawbacks to traditional tissue biopsy, such as its invasiveness, difficulties in repeat sampling, and implantation metastasis occurrence. As a noninvasive and safe approach, fluid biopsy brings a new perspective for tumor screening and diagnosis. Fluid biopsy provides a genetic snapshot of the tumor landscape, including both primary and metastatic loci [60].

TC is the most common cancer in American adults, aged between 16 and 33 years [61]. With a low fatality rate, but high and increasing incidence, the overdiagnosis of TC frequently occurs [38, 62, 63]. Routine diagnosis of thyroid nodules usually relies on ultrasound-mediated fine-needle aspirate biopsy (FNAB), which is invasive and often inaccurate Two challenges exist in TC diagnosis: (1) the identification of malignant thyroid nodules and benign nodules. Thyroid nodules are one of the most common symptoms in thyroid disease; approximately 15% of thyroid nodules are malignant [64]. Traditional methods guided by ultrasound [65] give rise to misdiagnoses, causing frequent overdiagnoses and overtreatments [66]. (2) Confirmation of the TC stage involved with LNM. Therapy methods for TC depends on tumor metastasis progression. Up to 50% neck-lymph node involved in TC patients and half of those nodes may be misjudged by neck-ultrasound. Isolation of genetic materials from human fluid is a minimally invasive method, leaving liquid biopsy a stage in the clinical approach to TC diagnosis.

The protein profiling is challenging for its small size, low abundance, and heterogeneity [67]. Still few researches found protein expressed significantly different among TC patients could be serve as biomarkers applied to improve diagnostic accuracy noninvasively. Fucosylation is one of the most crucial types of glycosylation involved in carcinogenesis. Comparing glycoproteomic difference of human body between healthy controls and patients with PTC, studies investigated that 44 fucosylated and 59 nonfucosylated desialo-N-glycopeptides were differentially expressed in patients, the ratio of fucosylated to fucosylated structures for each N-glycopeptide (F/NF) were correspondingly upregulated or downregulated in PTC, suggesting the F/NF ratio is a novel diagnostic marker of PTC [68]. Lin et al. publicised that 15 proteins were well distinguished between patients with LNM and patients without LNM. Additionally, the attenuation of tumor growth when knocking down ISG15 with shRNA implied that ISG15 probably serves as a prognosis indicator in fluid biopsy of thyroid papillary microcarcinoma patients with LNM [69]. Pazaitou-Panayiotou et al. found that ratios of insulin-like growth factor (IGF-1) to adiponectin and IGF-1 to (adiponectin × IGF-binding protein 3) were positively associated with tumor size even though the level of these three proteins had no obvious difference among several histologic types of TC [70]. These results definitely present the probably use of proteins in fluid biopsy in TC.

Studies focus on miRNAs in TC body fluids hint that miRNAs are the most potential circulating biomarkers for cancer diagnosis. The diagnosis of PTC persistence or recurrence, which cause significant inconvenience and trouble in patients, might benefit from fluid biopsy. Thyroglobulin is considered to be a main biomarker for detecting persistent/recurrent thyroid cancer. MiRNAs hold remarkable promise as serum biomarkers for monitoring TC patients when thyroglobulin assay results are uninformative. Rosignolo et al. discovered that circulating levels of miR-146a-5p and miR-221-3p were downregulated after tumor resection and investigated these molecules in serum as potential biomarkers for the early noninvasive detection of persistent PTC [71]. Another study demonstrated that miR-222 and miR-146b were associated with PTC recurrence; miR-222 and miR-146b circulatory levels corresponded to the presence of PTC [41]. These data suggest that different miRNAs could be markers of PTC. The distinct expression of miR-95 and miR-190 in PTC patient serum shows that miR-190 is upregulated, whereas miR-95 is downregulated, suggesting that these molecules act as complementary biomarkers for the differential diagnosis of thyroid nodules [72]. Li et al. suggested that levels of plasma miR-25-3p and miR-451a were significantly decreased after tumor excision from PTC patients. ROC analyses showed that plasma miR-25-3p and miR-451a levels had sensitivities of 92.8% and 88.9%, and specificities of 68.8% and 66.7%, respectively, in distinguishing PTC from benign nodules [73]. Another study revealed that the upregulation of both miR-146b-5p and miR-146b-3p characterised PTC-classical-type (CT) and PTC-tall-cell variant (TCV), but not PTC-follicular-variant (FV), whereas miR-21-5p was significantly overexpressed in PTC-TCV only. Also, miR-204-5p was downregulated in all histological subtypes of PTC-FV, except PTC-encapsulated follicular variant (EFV) [74]. These results demonstrate novel perspectives for the molecular classification of PTC, showing that different miRNA expression profiles are associated with different PTC histological types. Clearly, miRNA levels in the bloodstream of TC patients are fluctuant, indicating enormous potential for TC diagnostics and therapeutics.

#### 3.3.3. Exosome-Based Fluid Biopsy in TC

Exosomes exist in almost all fluids of the human body, such as plasma, serum, and urine. Due to their predominant status in blood flow, exosomes show tremendous advantages when compared with CTCs and ctDNA [75]. They exhibit their effects not only in pathogenic processes but also in therapeutic processes where a huge potential has been revealed. It has been reported that exosomes are now important fluid biopsy tools in several diseases, such as infections [21] and cancers like ovarian [76], prostate [77], gastric [78], breast [79], and brain tumours [80]. Although few research studies have been reported on exosomes released by thyroid cancer, experimental evidence exists to support the involvement of exosome contents especially miRNAs in thyroid cancers, showing the hypothesis that exosomes serve as fluid biopsy tools, for diagnosing and identifying different TC types.

Wang et al. investigated the upregulation of miR-346, miR-10a-5p, and miR-34a-5p in PTC plasma exosomes, establishing a three-miRNA plasma panel with huge potential clinical benefits in discriminating PTC from healthy controls, or nodular goiter [44]. Boufraqech M et al. reported that screening decreased exosomal miR-145 level of PTC and ATC could be regarded as an effective test in patients with inconclusive FNABs [52]. Comparing miRNA profiles of patients with recurrent PTC and those without recurrence, Lee et al. found that miRNA-146b and miRNA-222 in PTC-derived exosomes were overexpressed for about 10-fold, suggesting those miRNAs may be developed into alternative biomarkers for PTC recurrence [41, 81]. Exosomal miRNA profiling has made contributions to distinguishing PTC from follicular thyroid carcinoma (FTC). Studies have shown that miR-21-5p and miR-221-3p were upregulated in FTC-derived exosomes, while miR-181a was upregulated in PTC-derived exosomes. The ratio of miR-21-5p/miR-181a showed high sensitivity in identifying FPC and PTC. The combination of miR-21-5p/miR-181a and miR-221-3p/miR-181a ratios improves the specificity of identification. These observations indicate that the expression of miR--21-5p, miR-221-3p, and miR-181a has serious clinical applications for the diagnosis of differentiated TC [42] (Table 2).

More than a few cohort studies are necessitated to investigate exosomes as circulating biomarkers for TC diagnosis and treatment. Correlational studies are concentrated on exosomal miRNAs released by TCs. Up to now, investigations around other contents of exosomes may provide further evidence and strengthen the perspective in the future. Beyond all doubt, fluid biopsy will present huge capacity and superiority to play a critical role in therapeutic and diagnostic area in cancer diseases, bringing extraordinary benefits to clinical medicine to a large extent.

## 4. Exosomes in GD

GD is the most frequent thyroid goiter-related disease, accounting for 50–80% of hyperthyroidism [82]. Populations with GD mainly consist of females, aged between 30 and 50 years. GD comprises an enlarged and overactive thyroid gland and increased heart rate and proptosis [83]. Typical GD manifestation results from thyroid hormone overproduction, which is related to sensitised thyrotropin-receptor antibodies. GD is a chronic disease, similar to diabetes and heart disease, where long-term medical treatment is required, which is lack of therapies against primary pathogenic mechanisms [82]. GD diagnosis is relatively straightforward; nevertheless, therapeutic remedies are more perplexing. Despite great progress and development in current understanding of the cellular and molecular basis of autoimmunity, the current treatments for GD have been at best invariable over the last 50 years [84]. Typical treatments include antithyroid medications, radioactive iodine ablation, and surgery [85]. GD deserves further research as it seriously impacts the quality of life.

Connections between exosomes and GD pathogenesis have been investigated, since it was first discovered that exosomes are vehicles shuttling back and forth in various cells. Hiratsuka et al. collected exosomes from intractable GD patients, which could stimulate mRNA expression for IL-1, TNF-ɑ, and IL-6 in peripheral blood mononuclear cells compared with GD patients in remission or healthy controls, thereby activating immune reactions. This finding suggested that exosomes may play roles in GD pathogenesis [86].

In healthy individuals, the proliferation of thyroid cells and synthesis and secretion of thyroid hormones are regulated by a combination of TSH and its receptors. However, autoantibodies targeting the TSHR, mimic the actions of TSH, causing thyroid hyperfunction in GD patients. Edo et al. provided evidence that TSHR is detected in exosomes, secreted from normal and cancerous thyroid cell lines, and may exert decoy effects by binding to autoantibodies, thus ameliorating autoantibody-mediated activation in GD patients [45]. Interestingly, another study by Rossi et al. revealed that dichlorodiphenyltrichloroethane (DDT)-induced formation and shift of exosomes containing TSHRs dislodged from thyroid cells could directly stimulate lymphocytes produced by TSH receptor-stimulating autoantibodies, leading to the development of GD [87]. These results appear to be contradictory; therefore, they require further investigation on the function of TSHRs loaded in exosomes. Probably, these observations are caused by diverse circumstances. For example, the effect of TSHR is likely to be completely conflicting under physiological and pathological conditions.

## 5. Summary and Perspectives

As a crucial component of the extracellular vascular matrix, exosomes exist almost everywhere in circulation. Since their discovery, this small particle has been enigmatic, attracting researchers to get to the bottom of their mysteries. From our unequivocal knowledge, these molecules are secreted by all kinds of cells and then released into circulation as vehicles, transferring messages/cargoes from original cells to the target site. The exosomal sorting mechanisms as well as the fluctuant level of components appear to numerous scholars looking for their eternal secrets. Dramatically, exosomes emerged with dual characters. On the pernicious side, along with the delivery of exosomes, detrimental productions, such as inflammatory factors from pathogens and metabolites from necrotic tissues, interacted with target cells. Acting as a quiet accomplice, exosomes trigger further injuries in human body. Majority of researches focused on exosomal miRNAs and protein levels which turned out significantly variation during pathogenesis, opening a window for deciphering diseases mechanisms. Furthermore, blocking exosomal secretion from patient cells and transferring pathways of these cargos offer a breakthrough for disease therapy.

Alternatively, exosomes display antipathogenic activities, accompanied by releasing factors alleviating tumour development. Nevertheless, the majority of these pathways have not been unearthed, which demands more attention. Additionally, increasing discovered evidence concerning exosomes to be used as delivery vehicles for disease treatment indicates a huge potential for therapeutic areas. Undoubtedly, the enormous prospective of exosomes deserves additional investigation.

Thyroid-related diseases primarily consist of TC, thyroid goiter, and thyroid dysfunction, which include hypothyroidism and hyperthyroidism. Thyroid dysfunction can be dexterously diagnosed by clinical features and laboratory data, and further therapies are regularly intervened by medicine controlling. On the contrary, challenges exist in the stage and diagnosis of thyroid goiter and carcinoma. In addition, pathogenic and aggravating mechanisms of GD are still unclear. The function of exosomes brings light to these complexities. Recently, data showed that cargoes encapsulated in exosomes appeared to vary in patients with thyroid disease. Accordingly, several studies about the pathway of changing mechanisms revealed partial causes, thereby inducing further imagination about the application of exosomes. Most of those variation is significantly related to disease progression and prognosis, giving clues for diagnosis and treatment of thyroid diseases. Altered level of exosomal cargos correspond to distinctive pathological stage. Combination of several biomarkers makes TC diagnosis and treatment more conclusiveness.

Fluid biopsy is a novel technique which exhibits momentous potential for thyroid disease diagnosis and therapy. Even though miscellaneous, other irrelevant fluid components existing in circulation may cause instability to the detection results. Several liquid biopsy companion diagnostic tests approved by the regulatory agencies for the selection of patients in clinical practice light on an innovative way for fluid biopsy. In recent decades, tremendous advances and progress have been made in probing exosomes as regulators of thyroid disease. Incontrovertibly, the combination of exosomes and fluid biopsy creates a perspective for TC therapeutic field. However, up-to-date results are notwithstanding at the tip of the research iceberg. With advances in technology and more controlled, large-scale, and cohort-driven studies, unravelling the promise of exosomes to play its therapeutic potential for thyroid disease is just around the corner.

## Figures and Tables

**Table 1:** Role of exosomal contents in TC development.

Exosomal origin	Author	Contents	Function	Level	Reference
Serum	Luo et al.	SRC	Regulate tumor metastasis via activating MAPK/ERK and focal adhesion kinase (FAK) pathways	Overexpress	[46]

Hypoxic PTC cells	Wu et al.	miRNA-21-5p	Via miRNA-21-5p/TGFBI and miRNA-21-5p/COL4A1 pathway to promote angiogenesis of HUVECs	Overexpress	[47]

PTC serum	Ye et al.	miRNA-432-5p	Accelerating the lymph node metastasis	Overexpress	[48]

TC-derived cell lines	Hardy	SNAIL	Repressing CDH1 transcription and promoting the development of PTC	Overexpress	[49]

CSCs	Hardin	Linc-ROR	Trigger EMT and induce the local tumor microenvironment and the distant metastatic niche	Overexpress	[36]

TPC-1 cell lines and 8505 cells	Boufraqech	miR-145	Via inhibiting the PI3K/Akt pathway to suppress the growth and transfer of TC	Downexpress	[52]

**Table 2:** Potential biomarkers of exosomal miRNAs in different types of TC.

Origin	Potential biomarker	Expression level	Reference
PTC plasma exosomes	miR-346, miR10a-5p, and miR34a-5p	Up	[44]
PTC- and ATC-derived exosomes	miR-145	Down	[52]
PTC-derived exosomes	miRNA-146b and miRNA-222	Up	[41, 81]
FTC-derived exosomes	miR-21-5p and miR-221-3p	Up	[42]
PTC-derived exosomes	miR-181a	Up	[42]

## Abbreviations

EVs: Extracellular vehicles
miRNA: MicroRNA
lncRNA: Long noncoding RNA
circRNA: Circular RNA
ESCRT: Endosomal sorting complex responsible for transport
MVEs: Multivesicular endosomes
DC: Dendritic cells
HUVEC: Human umbilical vein endothelial cell
HSPGs: Heparan sulfate proteoglycans
STAT3: Signal transducers and activators of transcription 3
TC: Thyroid cancer
PTC: Papillary thyroid cancer
MTC: Medullary thyroid cancer
ATC: Anaplastic thyroid cancer
GD: Grave disease
MAPK: Mitogen-activated protein kinases
PI3K–AKT: Phosphatidylinositol-3-kinase and protein kinase B
circRNAs: Circular RNAs
mTOR: Mammalian target of rapamycin
TSHR: Thyrotropin receptor
SPEs: Serum-purified exosomes
LNM: Lymph node metastasis
EMT: Epithelial-mesenchymal transitions
CSC: Cancer stem-like cell
VEGF: Vascular endothelial growth factor
FAK: Focal adhesion kinase
ctDNA: Circulating tumor DNA
CTCs: Circulating tumor cells
FTC: Follicular thyroid carcinoma
IGF-1: Insulin-like growth factor
FNAB: Fine-needle aspirate biopsy.
